# Supplementary material for: Analytic modelling of a planar Goubau line with circular conductor
Source: Sci Rep. 2020 Nov 27;10:20754. doi: 10.1038/s41598-020-77703-w (PMC7695854; doi:10.1038/s41598-020-77703-w)
Supplement: Supplementary file 1 — Supplementary Information. [file 41598_2020_77703_MOESM1_ESM.pdf]

Supplementary Material For: Analytic Modelling of a Planar Goubau Line with Circular Conductor

Tobias Schaich[1,*], Daniel Molnar[1], Anas Al Rawi[1,2], and Mike Payne[1]

[1]University of Cambridge, Department of Physics, Cambridge, CB3 0HE, UK
[2]BT Labs, Adastral Park, Orion building, Martlesham Heath, Ipswich, IP5 3RE, UK
[*]Corresponding Author Email: tcs49@cam.ac.uk

S1 Numerical Model

Finite Element Calculations To model surface wave propagation on the planar Goubau line (PGL) numerically, we utilize the commercial finite element method (FEM) code COMSOL Multiphysics. The frequency domain Helmholtz equation is solved, where the $e^{i\omega t}$ dependence is assumed throughout. By solving this equation the out-of-plane component of the electric field is obtained and the remaining field components are calculated from Maxwells equations. We obtain the longitudinal complex propagation constant (β) of the out-of-plane z direction, which has real part ν, and complex part α which signifies the longitudinal attenuation.

The Helmholtz vector equation for the electric field $\vec{E}$ solved in COMSOL is given by:

$$\vec{\nabla} \times \mu_r^{-1}(\vec{\nabla} \times \vec{E}) - k_0^2(\varepsilon_r - \frac{i\sigma}{\epsilon_0 \omega})\vec{E} = \vec{0}$$

with angular frequency ω, free-space propagation constant k_0 and permittivity of the vacuum ϵ_0. Material parameters such as the relative dielectric constant ε_r, conductivity σ and relative permeability μ_r are defined in the geometrical model of the system under consideration.

Domain and Boundary Conditions The boundary conditions applied when solving the Helmholtz equation in the FEM approach are the following (cf. Supplementary Figure 1). In the outer boundary if the radius of the circular air region surrounding the wire R is chosen large enough, it could either be a perfect electric conductor (PEC, $\vec{n} \times \vec{E} = 0$) or perfect magnetic conductor (PMC, $\vec{n} \times \vec{H} = 0$). However, in practice R is limited by the computational resources and a low reflecting boundary condition or a perfectly matched layer (PML) can be chosen to mimic infinite space. In this work we chose the latter. The outer most boundary of the PML is chosen to be PEC, but PMC could equivalently

suffice. The wire is PEC. For the substrate width w, we chose a sufficiently large value so that edge effects are negligible. In this case 800mm was sufficient.

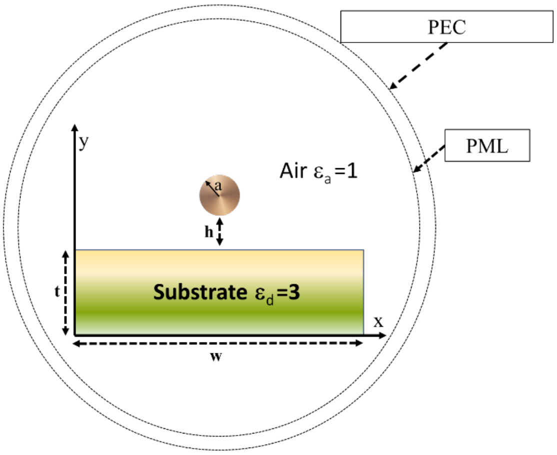

Supplementary Figure 1: Cross section schematics to show the boundary conditions used for single wires in this work. The coordinate system origin with respect to the substrate and wire is also shown.

The mesh element size is chosen according to the Shannon-Nyquist sampling condition as well as to resolve the smallest geometry features in the model. In our case using second order mesh element shape functions, it translates to a maximum element size of $\lambda/6$, where λ is the free space wavelength.

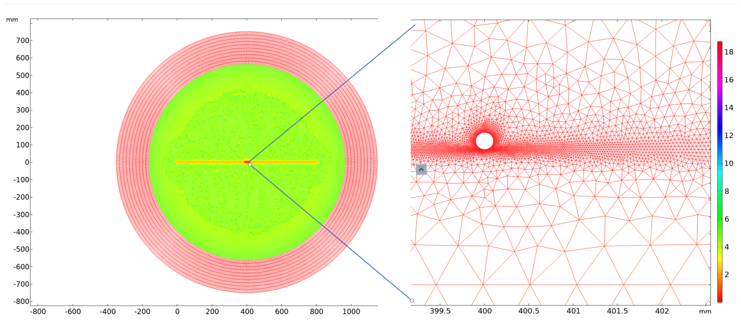

Supplementary Figure 2: Mesh for this present model. The elements are coloured by the mesh size according to the scale in mm. The enlarged region shows the mesh in the vicinity of the wire.

At 10 GHz it is around 5mm. However, since the wire shape needs to be resolved accurately the smallest size is ultimately set by the geometry constraint in our case, that is the wire radius of 0.1mm or more precisely a fraction of that. We found that by having 20 mesh elements for each quadrant of the circular cross section of the wire, therefore having 80 elements in total around the wires perimeter, the resolution is sufficient. As it can be seen in Supplementary Figure 2, COMSOLs meshing algorithm automatically grows the element size from this to the required $\lambda/6$ in the uniform, air domain surrounding the wire, but this

growth rate can be made fully manually controlled. For the PML, because of the underlying coordinate stretching formula, a special mesh type a mapped mesh is used. Otherwise standard triangular elements suffice throughout the model. The colouring scheme is according to the mesh element size of the sides of the triangle (or the quadraliteral elements in the PML) measured in mm.

S2 Matrix $\underline{\underline{\mathbf{Q}}}$

$$\underline{\underline{Q}} = \begin{pmatrix} -1 & 1 & 1 & 0 & 0 & 0 & 0 & 0 \\ 0 & e^{-iu_2 b} & e^{iu_2 b} & -e^{iu_1 b} & 0 & 0 & 0 & 0 \\ 0 & 0 & 0 & 0 & 1 & -1 & -1 & 0 \\ 0 & 0 & 0 & 0 & 0 & e^{-iu_2 b} & e^{iu_2 b} & -e^{iu_1 b} \\ -\frac{k_1\sqrt{\varepsilon_{r,1}}u_1}{\gamma_1^2} & \frac{k_2\sqrt{\varepsilon_{r,2}}u_2}{\gamma_2^2} & -\frac{k_2\sqrt{\varepsilon_{r,2}}u_2}{\gamma_2^2} & 0 & -\frac{\beta\xi}{\gamma_1^2} & \frac{\beta\xi}{\gamma_2^2} & \frac{\beta\xi}{\gamma_2^2} & 0 \\ 0 & \frac{k_2\sqrt{\varepsilon_{r,2}}u_2}{\gamma_2^2}e^{-iu_2 b} & -\frac{k_2\sqrt{\varepsilon_{r,2}}u_2}{\gamma_2^2}e^{iu_2 b} & \frac{k_1\sqrt{\varepsilon_{r,1}}u_1}{\gamma_1^2}e^{iu_1 b} & 0 & \frac{\beta\xi}{\gamma_2^2}e^{-iu_2 b} & \frac{\beta\xi}{\gamma_2^2}e^{iu_2 b} & -\frac{\beta\xi}{\gamma_1^2}e^{iu_1 b} \\ -\frac{\beta\xi}{\gamma_1^2} & \frac{\beta\xi}{\gamma_2^2} & \frac{\beta\xi}{\gamma_2^2} & 0 & \frac{k_0 u_1}{\gamma_1^2} & -\frac{k_0 u_2}{\gamma_2^2} & \frac{k_0 u_2}{\gamma_2^2} & \\ 0 & \frac{\beta\xi}{\gamma_2^2}e^{-iu_2 b} & \frac{\beta\xi}{\gamma_2^2}e^{iu_2 b} & -\frac{\beta\xi}{\gamma_1^2}e^{-iu_1 b} & 0 & -\frac{k_0 u_2}{\gamma_2^2}e^{-iu_2 b} & -\frac{k_0 u_2}{\gamma_2^2}e^{iu_2 b} & -\frac{k_0 u_1}{\gamma_1^2}e^{iu_1 b} \end{pmatrix}$$

S3 Function $F(\xi)$

$$\begin{gathered} F(\xi) = 2\gamma_2^2 k_0^2 u_1 \Big[-\beta^2(-1+e^{2iu_2 b})(\gamma_1^2-\gamma_2^2)^2\xi^2(\gamma_2^2 u_1+\gamma_1^2 u_2) - \\ k_0(\gamma_2^2 k_0 u_1 + \sqrt{\varepsilon_{r,2}}\gamma_1^2 k_2 u_2)\Big((-1+e^{2ibu_2})\gamma_2^4 u_1^2 - 2(1+e^{2ibu_2})\gamma_1^2\gamma_2^2 u_1 u_2 + \\ (-1+e^{2ibu_2})\gamma_1^4 u_2^2\Big) + e^{2ibu_2}\Big(-\beta^2(-1+e^{2ibu_2})(\gamma_1^2-\gamma_2^2)^2\xi^2(-\gamma_2^2 u_1+\gamma_1^2 u_2) + \\ k_0(\gamma_2^2 k_0 u_1 - \sqrt{\varepsilon_{r,2}}\gamma_1^2 k_2 u_2)((-1+e^{2ibu_2})\gamma_2^4 u_1^2 - 2(1+e^{2ibu_2})\gamma_1^2\gamma_2^2 u_1 u_2 \\ +(-1+e^{2ibu_2})\gamma_1^4 u_2^2))\Big)\Big] / \Big[\beta^4(-1+e^{2ibu_2})^2(\gamma_1^2-\gamma_2^2)^4\xi^4 + \\ 2\beta^2(-1+e^{2ibu_2})(\gamma_1^2-\gamma_2^2)^2 k_0\xi^2((-1+e^{2ibu_2})\gamma_2^4 k_0 u_1^2 - \\ (1+e^{2ibu_2})\gamma_1^2\gamma_2^2(k_0+\sqrt{\varepsilon_{r,2}}k_2)u_1 u_2 + (-1+e^{2ibu_2})\sqrt{\varepsilon_{r,2}}g_1^4 k_2 u_2^2) + \\ k_0^2((-1+e^{2ibu_2})\gamma_2^4 u_1^2 - 2(1+e^{2ibu_2})\gamma_1^2\gamma_2^2 u_1 u_2 + (-1+e^{2ibu_2})\gamma_1^4 u_2^2) \\ \Big((-1+e^{2ibu_2})\gamma_2^4 k_0^2 u_1^2 - 2(1+e^{2ibu_2})\sqrt{\varepsilon_{r,2}}\gamma_1^2\gamma_2^2 k_0 k_2 u_1 u_2 + \\ (-1+e^{2ibu_2})\varepsilon_{r,2}\gamma_1^4 k_2^2 u_2^2\Big)\Big] \end{gathered}$$
